# Functional abnormalities in iPSC-derived cardiomyocytes generated from CPVT1 and CPVT2 patients carrying ryanodine or calsequestrin mutations

**DOI:** 10.1111/jcmm.12581

**Published:** 2015-07-08

**Authors:** Atara Novak, Lili Barad, Avraham Lorber, Mihaela Gherghiceanu, Irina Reiter, Binyamin Eisen, Liron Eldor, Joseph Itskovitz-Eldor, Michael Eldar, Michael Arad, Ofer Binah

**Affiliations:** a Department of Physiology, TechnionHaifa, Israel; b The Rappaport Institute for Research in the Medical Sciences, TechnionHaifa, Israel; c Ruth & Bruce Rappaport Faculty of Medicine, TechnionHaifa, Israel; d Department of Pediatric Cardiology, Rambam Health Care CampusHaifa, Israel; e ‘Victor Babes’ National Institute of PathologyBucharest, Romania; f Department of Plastic Surgery, Rambam Health Care CampusHaifa, Israel; g Leviev Heart Center, Sheba Medical Center, Tel Hashomer and Sackler School of Medicine, Tel Aviv UniversityTel Aviv, Israel

**Keywords:** catacholaminergic polymorphic ventricular tachycardia, Induced pluripotent stem cells, arrhythmias, cardiomyocytes, Ca^2+^ transients

## Abstract

Catecholaminergic polymorphic ventricular tachycardia (CPVT) is an inherited arrhythmia characterized by syncope and sudden death occurring during exercise or acute emotion. CPVT is caused by abnormal intracellular Ca^2+^ handling resulting from mutations in the RyR2 or CASQ2 genes. Because CASQ2 and RyR2 are involved in different aspects of the excitation-contraction coupling process, we hypothesized that these mutations are associated with different functional and intracellular Ca²^+^ abnormalities. To test the hypothesis we generated induced Pluripotent Stem Cell-derived cardiomyocytes (iPSC-CM) from CPVT1 and CPVT2 patients carrying the RyR2^R420Q^ and CASQ2^D307H^ mutations, respectively, and investigated in CPVT1 and CPVT2 iPSC-CM (compared to control): (*i*) The ultrastructural features; (*ii*) the effects of isoproterenol, caffeine and ryanodine on the [Ca^2+^]_i_ transient characteristics. Our major findings were: (*i*) Ultrastructurally, CASQ2 and RyR2 mutated cardiomyocytes were less developed than control cardiomyocytes. (*ii*) While in control iPSC-CM isoproterenol caused positive inotropic and lusitropic effects, in the mutated cardiomyocytes isoproterenol was either ineffective, caused arrhythmias, or markedly increased diastolic [Ca^2+^]_i_. Importantly, positive inotropic and lusitropic effects were not induced in mutated cardiomyocytes. (*iii*) The effects of caffeine and ryanodine in mutated cardiomyocytes differed from control cardiomyocytes. Our results show that iPSC-CM are useful for investigating the similarities/differences in the pathophysiological consequences of RyR2 *versus* CASQ2 mutations underlying CPVT1 and CPVT2 syndromes.

## Introduction

Catecholaminergic polymorphic ventricular tachycardia (CPVT) is a familial arrhythmogenic disorder characterized by episodic syncope and sudden death occurring during exercise. CPVT is caused by abnormal Ca^2+^ handling resulting from an autosomal dominant mutation in the RyR2 gene (CPVT1) or the autosomal recessive mutation in the CASQ2 genes (CPVT2) 1–3. Briefly, RyR2 is a Ca²^+^ release sarcoplasmic reticulum (SR) channel with a key role in the Calcium-Induced-Calcium-Release during the excitation contraction (E-C) coupling. CASQ2 is a high-capacity, low affinity Ca²^+^ binding protein, operating as a major Ca²^+^ buffering factor. Since both proteins are major players in (E-C) coupling, the functional derangements in intracellular Ca²^+^ handling resulting from the mutated RyR2 and CASQ2 may cause delayed afterdepolarizations (DADs) and triggered arrhythmias which constitute the electrophysiological mechanism underlying CPVT 1–3. Recently, induced pluripotent stem cell (iPSC)-derived cardiomyocytes (iPSC-CM) from CPVT patients demonstrated our ability to establish disease-specific cardiomyocytes that recapitulate the clinical phenotype [*e.g*. 4–8]. Hence, our group was the first to generate iPSC-CM from CPVT2 patients carrying the CASQ2^D307H^ mutation, and to demonstrate that the mutated cardiomyocytes generated DADs and triggered arrhythmia in response to β-adrenergic stimulation 7. Because CASQ2 and RyR2 have different roles in the E-C coupling, we hypothesized that mutations in these genes will cause different functional Ca²^+^ handling abnormalities. We tested the hypothesis by comparing in CPVT1 and CPVT2 iPSC-CM: (*i*) The ultrastructure; (*ii*) the effects of isoproterenol, caffeine and ryanodine on the [Ca^2+^]_i_ transient characteristics. Briefly, the mutated cardiomyocytes were less developed untrastructurally than control, and the responses to isoproterenol, caffeine and ryanodine were different than control. These findings demonstrate that the mutated iPSC-CM are useful for investigating the similarities/differences in the pathophysiological consequences of RyR2 *versus* CASQ2 mutations underlying CPVT1 and CPVT2.

## Materials and methods

### Generation of iPSC, genotyping and karyotype analysis

Skin biopsies were obtained from a 25-years old CPVT1 woman (HDF15) carrying RyR2^R420Q^ mutation and from 4 CPVT2 patients carrying the CASQ2^D307H^ mutation, as described in the Supplement. Of the 4 CPVT patients, 2 patients (Clones 7.5, 7.6 and 12.4) were included in our previous study 7, and 2 patients (Clones 19.1, 19S1, 20S3 and 20.1) have not been previously investigated. As controls, we used a skin biopsy from a 46 years old volunteer (HDF24) and hair keratinocytes from the plucked hairs of 2 healthy volunteers at the ages of 36 (KTN) and 41 (KTR), as described in the Supplement 9. Details regarding iPSC generation, the different clones employed, and the immunofluorescence analysis are described in the Supplement.

### Measurements of [Ca^2+^]_i_ transients

[Ca^2+^]_i_ transients were measured from small contracting areas of EBs by means of fura-2 fluorescence, and analysed as described in the Data S1. At the beginning of each experiment we first measured the spontaneous beating rate. Following this measurement, all reparations were paced throughout the entire experiment (see details in the Data S1).

### Transmission electron microscopy

Transmission electron microscopy (TEM) was performed on 60–62 days-old (post-plating) EBs as described in the Supplement. The number, lengths (between two Z bands) and widths (at Z band level) of sarcomeres were measured in 15 cells with visible nucleus on section. The sarcoplasmic reticulum (SR) width and the number of RyR were measured on 25 TEM images. The results were presented as mean ± SEM and the significance of difference between the groups was determined using one-way anova followed by Dunn’s test.

### Statistical analysis

See Data S1 for details.

## Results

### Molecular and genetic characterization of CPVT1 and CPVT2 iPSC, and differentiation of iPSC into functional cardiomyocytes

**Figure 1:**
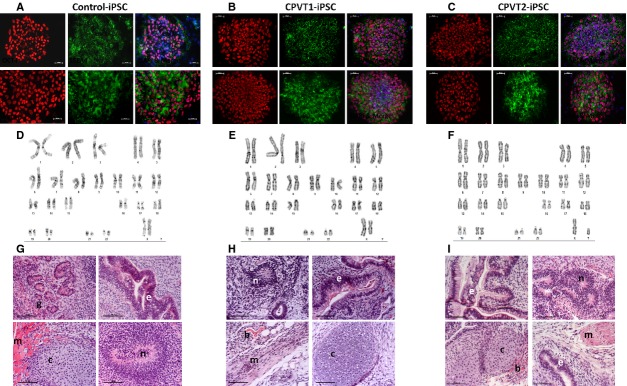
Pluripotency of iPSC derived from CPVT1 RyR2^R420Q^ and CPVT2 CASQ2^D307H^ patients and healthy control. (A–C) Immunostaining of typical pluripotent markers shown for iPSCs derived from (A) healthy control clone HDF24.2, (B) CPVT1 patient, clone HDF15.4 and (C) CPVT2 patient, clone 20.1. Nuclei were stained with DAPI (blue), scale bar 50 μm. (D–F) Karyotype analysis of iPSC derived from (D) healthy control clone HDF24.2, (E) CPVT1 patient clone HDF15.4 and (F) CPVT2 patient clone 20.1. (G–I) Histological analysis of a representative teratoma obtained from *in vivo* differentiated cells of (G) control iPSC-24S9, (H) CPVT1 iPSC-15.4 and (I) CPVT2 iPSC-20.1. The formed teratomas contained derivatives of all three germ layers (ectoderm, mesoderm and endoderm). Mesoderm: (m) muscle, (c) cartilage, (b) blood cells. Endoderm: (e) epithelium, (g) endocrine glands. Ectoderm: (n) neural rosette. All images were obtained from formalin-fixed and paraffin-embedded teratoma sections stained with haematoxylin and eosin, scale bar 50 μm.

**Figure 2:**
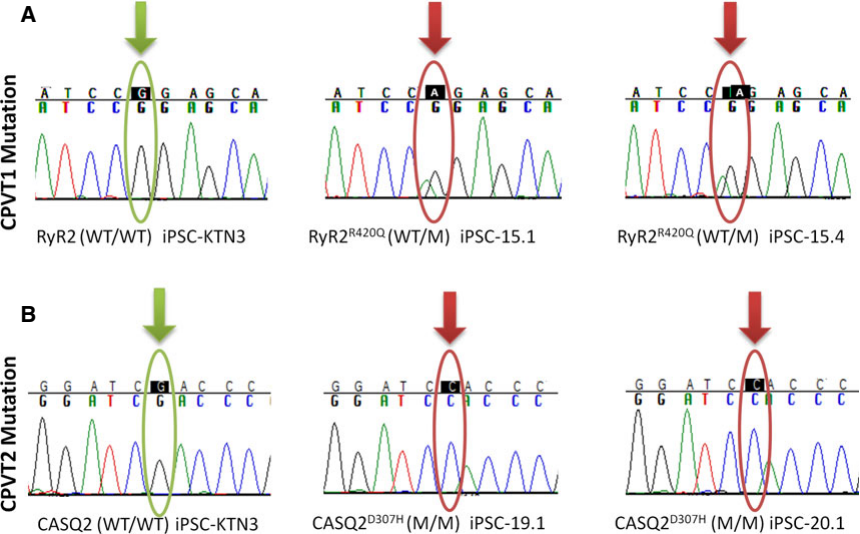
Genotyping the CPVT1 RyR2 R420Q mutation and CPVT2 CASQ2 D307H mutation. (A) Sequence chromatograms from the CPVT1 iPSC-HF15.1 and 15.4 clones (WT/M), and from a healthy non-carrier iPSC-KTN3 clone (WT/WT). The analysis shows a *G* to *A* substitution at nucleotide 1378 in exon 13, converting an arginine amino acid to glutamine at position 420 of the protein. (B) Sequence chromatograms from the CPVT2 iPSC-HDF19.1, 20.1 clones (M/M) and a healthy non-carrier iPSC-KTN3 clone (WT/WT). There is a *G* to *C* substitution at nucleotide 1183 in exon 9, converting aspartic acid to histidine at codon 307.

**Figure 3:**
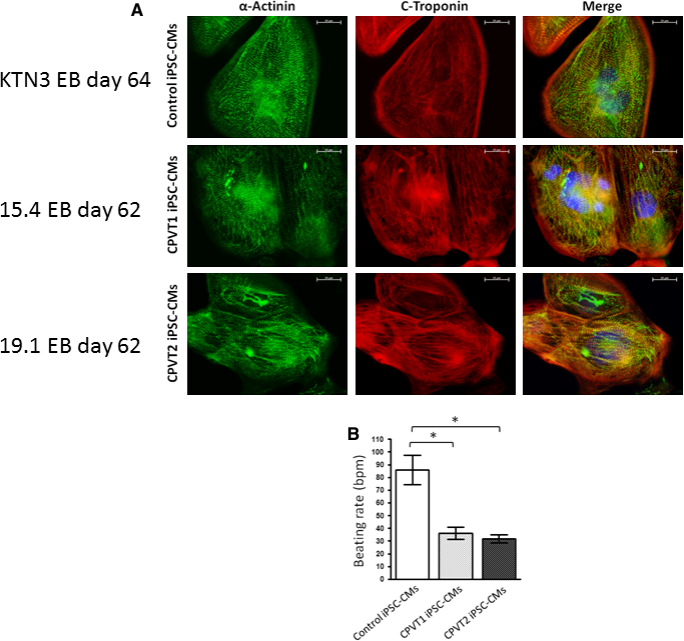
Cardiac differentiation of iPSC-derived from CPVT1 RyR2^R420Q^ and CPVT2 CASQ2D307H patients and healthy control. (A) Immunofluorescence expression of typical myofilament in cardiomyocytes (62–64 days old) of control (clone KTN3), CPVT1 (clone 15.4) and CPVT2 (clone 19.1) stained for the typical myofilament proteins; α-actinin and cardiac troponin, scale bar 10 μm. (B) Spontaneous beating rate of control-CM (*n* = 9), CPVT1-CM (*n* = 10) and CPVT2-CM (*n* = 10), bpm: beats per minute. Statistical analysis of one-way anova followed by Dunn’s test, *P < 0.05.

All the clones we used expressed the pluripotent markers Oct4, Nanog, SSEA4 and TRA1-60 (Fig.1A–C), had normal karyotype (Fig.1D–F), and demonstrated pluripotency as illustrated by their ability to differentiate into endoderm, mesoderm and ectoderm (Fig.1G–I). Furthermore, using sequencing of the genomic PCR product we confirmed the switch of *G* to *A* at nucleotide 1378 in the RyR2 gene (Fig.2A) 10, and the switch of *G* to *C* at nucleotide 1183 in the CASQ2 gene (Fig.2B) 7,11. Next, control, CPVT1 and CPVT2 iPSC were spontaneously differentiated into functional cardiomyocytes 7,9. Immunofluorescence staining of micro-dissected contracting areas from EBs (Fig.3A) demonstrated that the cardiomyocytes co-express the typical cardiac markers, cardiac troponin C and α-sarcomeric actinin. Importantly, the cardiomyocytes exhibited areas of cross-striations, indicating organization towards myofibrilar structures. Finally, the spontaneous firing rates of CPVT1 and CPVT2 iPSC-CM were significantly (*P* < 0.01) lower than control iPSC-CM (Fig.3).

### Transmission electron microscopy analysis

**Figure 4:**
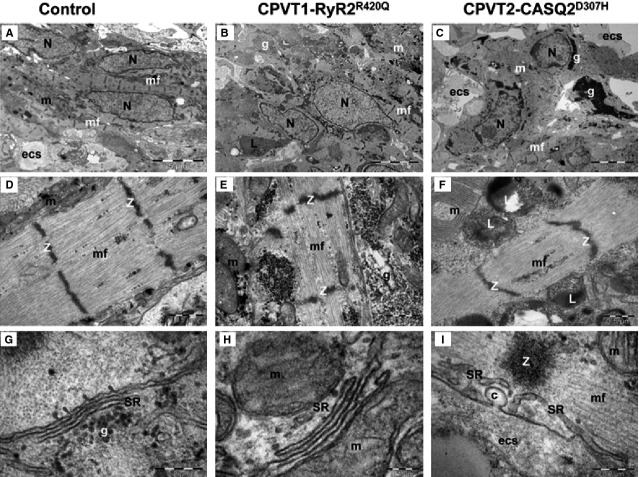
Electron microscopy of Control (A, D, G), CPVT1-RyR2^R420Q^ (B, E, H) and CPVT2-CASQ2^D307H^ (C, F, I) iPSC-CM. (A–C) show overall appearance of EBs. Nuclei (N), clusters of mitochondria (m), myofilaments (mf), glycogen masses (g), lysosomes (L) are visible in cytoplasm of cardiomyocytes. (D–F) Electron microscopy of the myofilaments (mf) organized in distinct sarcomeric structures defined by Z-bands. m - mitochondria, g - glycogen, L - lysosomes. (G–I) Electron microscopy of peripheral sarcoplasmic reticulum (SR) of cardiomyocytes. g: glycogen; m: mitochondria; mf: myofilaments; Z band, c: caveolae; ecs: extracellular space. Arrowheads indicate RyR between SR and sarcolemma.

**Figure 5:**
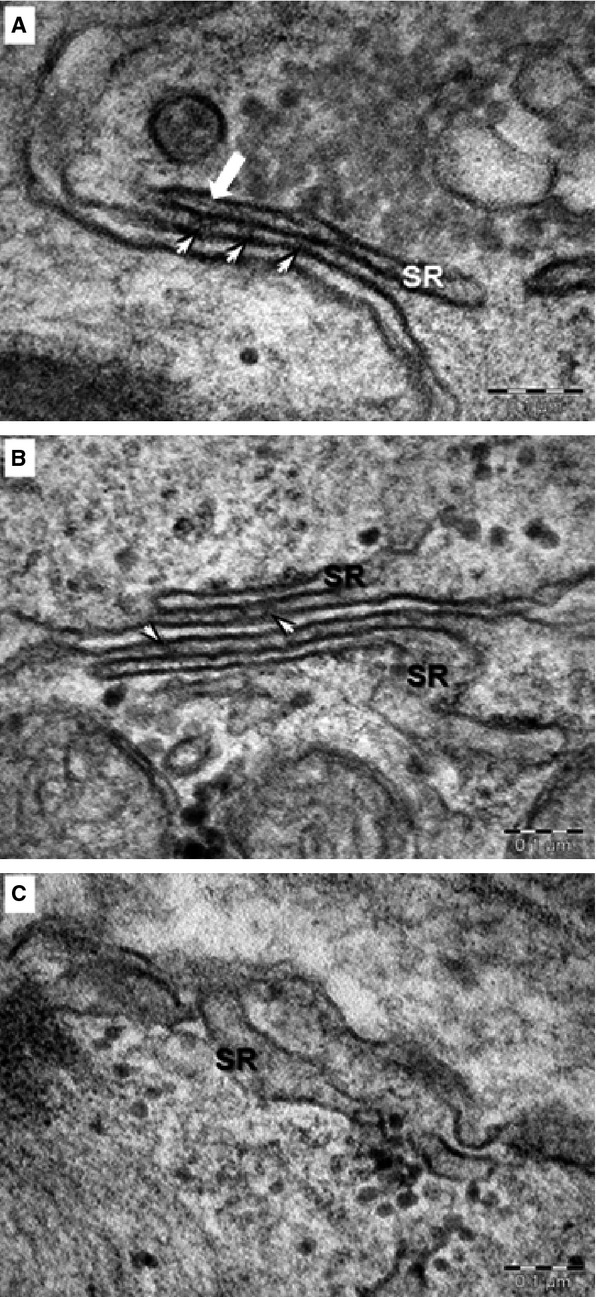
Electron microscopy of peripheral sarcoplasmic reticulum (pSR) in 60–62 days old control, clones KTN3 and HDF24.2 (A), CPVT1, clone 15.4 (B) and CPVT2, clones 20.1 and 19S1 (C) iPSC-CM. Ryanodine receptors (short arrows) are visible between pSR and sarcolemma in control (A) and CPVT1 (B) cardiomyocytes. Enlarged cisterna of pSR is seen in CPVT2 cardiomyocyte (B). Electron-dense linear structure formed by CASQ2 (thick white arrow) is visible within the SR lumen of control cardiomyocytes (A).

The ultrastructural analysis performed on 60–62 days old cardiomyocytes from control (clones KTN3 and HDF24.2), CPVT1 (clone 15.4) and CPVT2 iPSC-CM (clones 20.1 and 19S1, not included in our previous CPVT2 study) demonstrated that control (Fig.4A and D), CPVT1 (Fig.4B and E) and CPVT2 iPSC-CM (Fig.4C and F) differentiated into cardiomyocytes with immature features: myofibrils with variable degrees of organization in a sarcomeric pattern, high mass of glycogen and large amounts of mitochondria. The analysis further showed that control iPSC-CM myofibrils exhibit typical sarcomeric pattern (Fig.4) with parallel Z-bands lined up (Table1). Clusters of mitochondria, glycogen and rough endoplasmic reticulum were also noted. Sarcoplasmic reticulum cisternae were present between the parallel myofibrils and in the periphery of cardiomyocytes. Peripheral SR (pSR) showed uniform wide cisternae (Table1), and RyR complexes were visible as dense nanostructures (‘feet’) connecting the pSR and sarcolemma (Fig.5, Table1). Furthermore, small clumps of CASQ2 were visible in the lumen of few pSR cisternae in control iPSC-CM (Fig.5A). In contrast, CPVT1 iPSC-CM showed a more immature phenotype: large clusters of mitochondria, glycogen and lysosomes compared to control iPSC-CM (Fig.4). Compared to control (Table1) CPVT1 iPSC-CM had smaller number of sarcomeres/cell (*P* < 0.05) and Z-bands were slenderer (*P* < 0.05) and more disarrayed. Furthermore, the CPVT1 iPSC-CM pSR SR showed narrower cisternae (Fig.5B, Table1, *P* < 0.05), a much smaller number of ‘feet’ (Table1, *P* < 0.05), and no visible dense chain-like nanostructures were found within the pSR lumen. CPVT2 iPSC-CM exhibited the most immature phenotype with large masses of glycogen, clusters of mitochondria, lysosomes and disarrayed myofibrils (Fig.4). Specifically, CPVT2 iPSC-CM had smaller number of sarcomeres/cell than control (*P* < 0.05, Table1), and sarcomeres were narrower (*P* < 0.05). pSR often showed enlarged (*P* < 0.05) and empty cisternae (Fig.5C). In addition, uniform narrow cisternae containing fewer ‘feet’ (RyR) in contact with plasmalemma (Table1) were visible in CPVT2 iPSC-CM. Electron-dense chain-like nanostructures were undetected within the pSR lumen of CPVT2 iPSC-CM.

**Table 1:** Comparative analysis of structural and morphometric aspects of Control, CPVT1 and CPVT2 cardiomyocytes. The results are expressed as mean ± SD

	Control	CPVT1-RyR2^R420Q^	CPVT2-CASQ2^D307H^	*P*-value		
				C *versus* C1	C *versus* C2	C1 *versus* C2
Number of sarcomeres/cell	6.33 ± 0.77 (*n* = 15)	3.47 ± 0.47 (*n* = 15)	2.67 ± 0.40 (*n* = 15)	*P* < 0.05	*P* < 0.05	*N.S*
Sarcomere length (μm) [Z-Z distance]	1.66 ± 0.04 (*n* = 30)	1.57 ± 0.02 (*n* = 30)	1.51 ± 0.05 (*n* = 30)	*N.S*	*N.S*	*N.S*
Sarcomere width (μm) [Z band width]	1.26 ± 0.08 (*n* = 40)	0.52 ± 0.03 (*n* = 40)	0.57 ± 0.04 (*n* = 40)	*P* < 0.05	*P* < 0.05	*N.S*
The average width of the peripheral SR (nm)	22.84 ± 0.88 (*n* = 55)	19.35 ± 0.61 (*n* = 54)	26.13 ± 1.29 (*n* = 53)	*P* < 0.05	*N.S*	*P* < 0.05
Number of the RyR/μm	11.16 ± 0.79 (*n* = 31)	1.90 ± 0.57 (*n* = 6)	1.67 ± 0.58 (*n* = 7)	*P* < 0.05	*P* < 0.05	*N.S*

### Ca^2+^ handling abnormalities in mutated iPSC-CM

Because CPVT is an inherited arrhythmia resulting from increased cytoplasmic [Ca^2+^]_i_ we utilized three protocols to decipher the differences/similarities in the intracellular Ca^2+^ abnormalities in the mutated cardiomyocytes: β-adrenergic stimulation, exposure to caffeine and to ryanodine.

#### β-adrenergic stimulation

**Figure 6:**
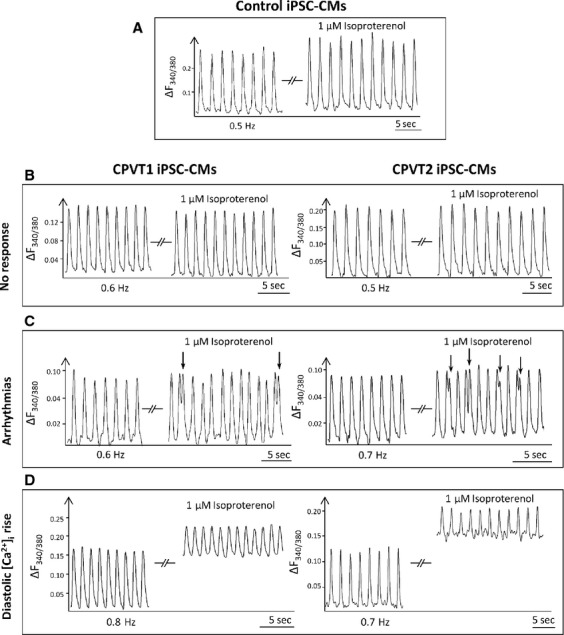
The effects of isoproterenol on the [Ca^+2^]_i_ transients in control, CPVT1and CPVT2 iPSC-CM. (A) [Ca^+2^]_i_ transients from control (clone 24.2 day 54) iPSC-CM paced at 0.5 Hz, in the absence and presence of isoproterenol. (B–D) Representative experiments in CPVT1 and CPVT2 iPSC-CM demonstrating the 3 types of responses to isoproterenol. Left side: CPVT1 (clones 15.4 day 56, 15.1 day 49, 15.1 day 38) iPSC-CM; Right side: CPVT2 iPSC-CM (clones 20.1 day 67, 19S1 day 43, 12.1 day 39). (B) No response to isoproterenol. (C) Isoproterenol induced triggered beats, marked by the arrows. (D) Isoproterenol increased diastolic [Ca^+2^]_i_. In (B–D) the pacing rates are shown in each panel. Arrhythmias are marked by black arrows.

**Figure 7:**
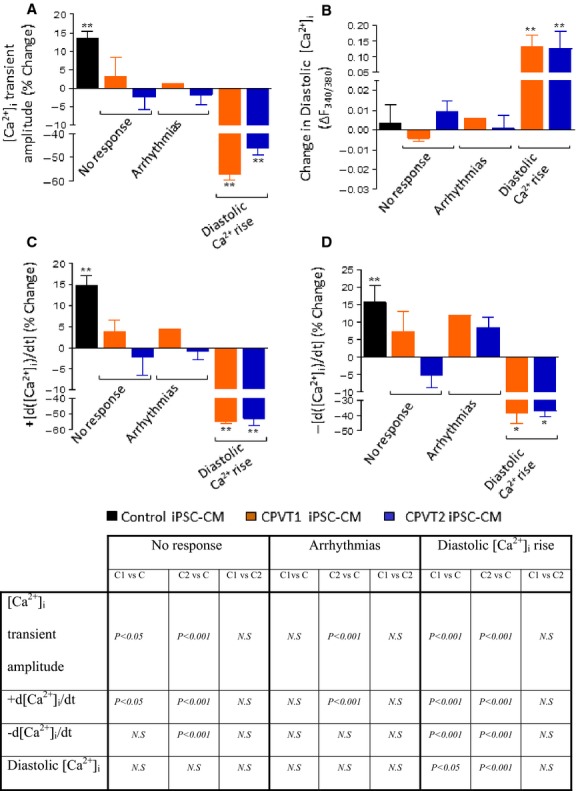
Summary of the effects of isoproterenol on the [Ca^2+^]_i_ transients of control, CPVT1 and CPVT2 iPSC-CM. (A) [Ca^+2^]_i_ transient amplitude; (B) Diastolic [Ca^2+^]_i_; (C) +d[Ca^2+^]_i_/dt; (D) −d[Ca^2+^]_i_/dt. In (A, C and D), the effect of isoproterenol was expressed as per cent change from control, and in (B), the effect was expressed as the change in the fluorescence ratio, Δ_340/380_, *P < 0.05, **P < 0.001. Asterisks above columns represent statistically significant effect of isoproterenol. The statistical analysis of differences among groups is shown in the Table below: C - Control iPSC-CM (*n* = 8), C1 - CPVT1 iPSC-CM (*n* = 12), C2 - CPVT2 iPSC-CM (*n* = 19).

Investigating the effects of isoproterenol on the [Ca^2+^]_i_ transients in control *versus* mutated iPSC-CM served as an important experimental tool to decipher intracellular Ca^2+^ abnormalities in the mutated cardiomyocytes. As shown by the representative experiments (Fig.6A and Fig. S1A) and the summary of these experiments (Fig.7), in control iPSC-CM (paced, *n* = 8) isoproterenol increased (as expected) the [Ca^2+^]_i_ transient amplitude, the maximal rate of rise and decay (+d[Ca^2+^]_i_/dt and −d[Ca^2+^]_i_/dt, respectively), while diastolic [Ca^2+^]_i_ was unchanged. In contrast, in CPVT1 and CPVT2 iPSC-CM isoproterenol caused three different types of effects: (*i*) ‘No-response’ to isoproterenol; (*ii*) ‘Arrhythmias’ and (*iii*) ‘Intracellular Ca^2+^ rise’ associated with diminished [Ca^2+^]_i_ transient amplitude. Hence, while the effects of isoproterenol in healthy iPSC-CM are those reported by us and others in previous studies 5,7, the effects in the mutated were ‘abnormal’ (three different responses). To strengthen the validity of these important findings, we demonstrate that these unexpected responses to isoproterenol were observed in different clones generated from different CPVT patients (Fig.6B–D and Fig. S1B–D). The reproducibility of the findings in different patients enabled us include in the summary figure (Fig.7) results from the entire CPVT cohort. Based on these findings, the effects of isoproterenol were analysed separately in these three groups (Fig.7). In summary: (*i*) No-response to isoproterenol: 59% in CPVT1 iPSC-CM (composed of 43% from clone 15.4 and 57% from clone 15.1); 37% in CPVT2 iPSC-CM (composed of 58% from clone 12.4, 14% from clone 7.14, 14% from clone 19S1 and 14% from clone 20.1. (*ii*) Arrhythmias: 16% in CPVT1 iPSC-CM clone 15.1; 37% in CPVT2 iPSC-CM (composed of 58% from clone 12.4), 14% from clone 12.12, 14% from clone 19S1 and 14% in clone 7.5. (*iii*) Marked intracellular Ca^2+^ rise associated with diminished [Ca^2+^]_i_ transient amplitude: 25% in CPVT1 iPSC-CM clone 15., and 26% in CPVT2 iPSC-CM (composed of 40% from clone 12.4, 20% from clone 7.5 and 20% from clone 20.1). Collectively, in both CPVT1 and CPVT2 iPSC-CM, in the ‘No-response’ group none of the [Ca^2+^]_i_ transient characteristics was affected, and arrhythmias were not generated. In the ‘Arrhythmias’ group none of the [Ca^2+^]_i_ transient characteristics was affected (Figs6 and 7). In the ‘Intracellular [Ca^2+^]_i_ rise’ group isoproterenol decreased (*P* < 0.05) the [Ca^2+^]_i_ transient amplitude, +d[Ca^2+^]_i_/dt and −d[Ca^2+^]_i_/dt.

#### Responsiveness to caffeine

**Figure 8:**
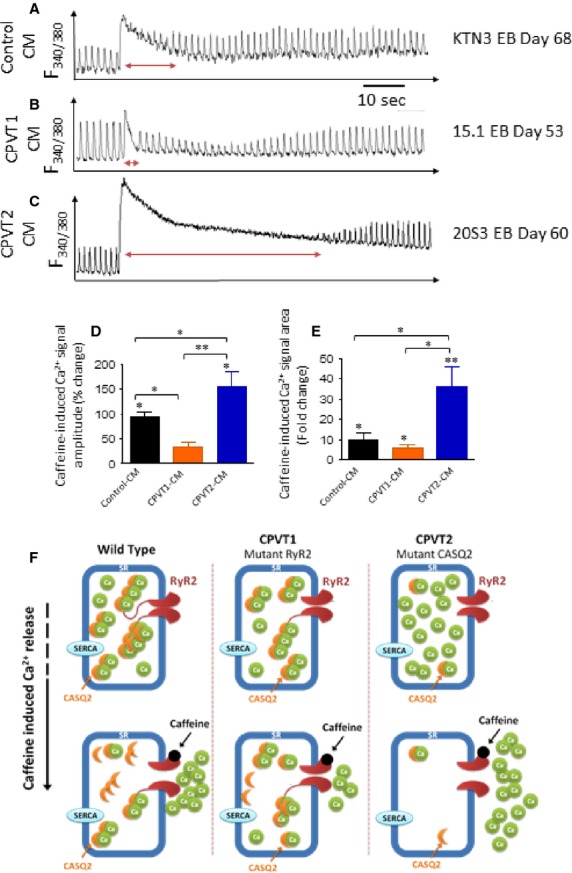
The effect of caffeine on the intracellular Ca^2+^ cycling of control, CPVT1 and CPVT2 iPSC-CM. (A–C) [Ca^2+^]_i_ transients from control (clone KTN3 day 68), CPVT1 (clone 15.1 day 53) and CPVT2 (clone 20S3 day 60) iPSC-CM, respectively, demonstrating the effect of caffeine. (D) The per cent change in caffeine-induced Ca^2+^ signal amplitude, compared to the pre-caffeine [Ca^2+^]_i_ transient amplitude. (E) The per cent change in area of the caffeine-induced Ca^2+^ signal, compared to the pre-caffeine [Ca^2+^]_i_ transient area. Control iPSC-CM (*n* = 7), CPVT1 iPSC-CM (*n* = 8), CPVT2 iPSC-CM (*n* = 8), *P < 0.05, **P < 0.001. Asterisks above columns represent statistically significant effect of caffeine, asterisk above bars connecting columns represent significant difference between groups. (F) A schematic model describing the effects of caffeine in the three groups. In control caffeine depletes SR free Ca^2+^ stores. Because in CPVT2 there is more (due to the mutated CASQ2) luminal free Ca^2+^ than in CPVT1 (due to ‘leaky’ RyR2), the response to caffeine is much more pronounced in CPVT2 than in control and CPVT1 iPSC-CM.

In these experiments, we investigated the Ca^2+^ storage/release capacities of the mutated *versus* control cardiomyocytes, by rapid application of 10 mM caffeine (an opener of the RyR2 receptor) to paced cardiomyocytes. As depicted in Figure8A, in control cardiomyocytes clone KTN3 caffeine caused an abrupt increase in intracellular Ca^2+^ along with a sharp decline in the [Ca^2+^]_i_ transient amplitude. Within 20 sec. after intracellular Ca^2+^ peaked, it declined and the [Ca^2+^]_i_ transients attained a steady state level close to the their pre-caffeine amplitude. Similar results are shown for clone 24.5 in Figure S2A. In CPVT1 cardiomyocytes clone 15.1 (Fig.8B) the response to caffeine was smaller and shorter, and the resumption of pre-caffeine intracellular Ca^2+^ level and [Ca^2+^]_i_ transient amplitude was much faster than in control cardiomyocytes. Similar results are shown for clone 15.4 in Figure S2B. In sharp contrast, in CPVT2 cardiomyocytes clone 20S3 (Fig.8C) the response to caffeine was markedly augmented, and the recovery time for both intracellular Ca^2+^ level and [Ca^2+^]_i_ transient amplitude was much longer, in the order of 50–60 sec. Similar results are shown for clone 19S1 in Figure S2C. To quantify the response to caffeine we calculated two parameters: (*i*) The per cent change in caffeine-induced Ca^2+^ signal amplitude, compared to the pre-caffeine [Ca^2+^]_i_ transient amplitude. (*ii*) The per cent change in the area of the caffeine-induced Ca^2+^ signal, compared to the pre-caffeine [Ca^2+^]_i_ transient area. Hence, compared to control cardiomyocytes, both measures of the caffeine response were smaller (*P* < 0.05) in CPVT1 cardiomyocytes and larger (*P* < 0.05) CPVT2 cardiomyocytes (Fig.8D–E).

#### Responsiveness to ryanodine

**Figure 9:**
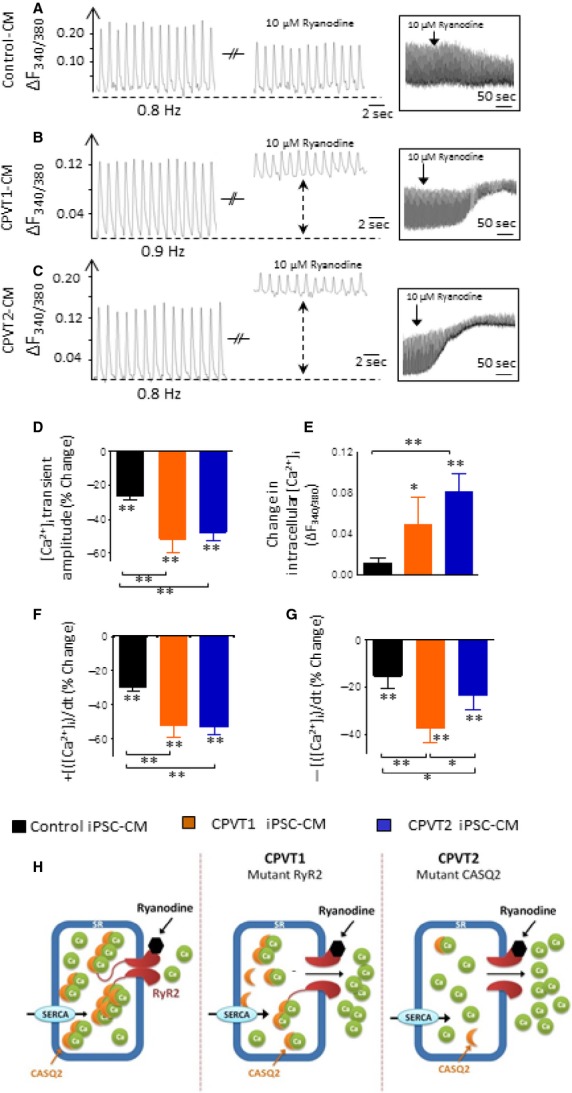
The effects of ryanodine on the [Ca^2+^]_i_ transients of control, CPVT1 and CPVT2 iPSC-CM. (A–C) [Ca^2+^]_i_ transients from the three groups demonstrating the effect of ryanodine. (D) [Ca^+2^]_i_ transient amplitude; (E) Diastolic [Ca^2+^]_i_; (F) +d[Ca^2+^]_i_/dt; (G) −d[Ca^2+^]_i_/dt. Control iPSC-CM (*n* = 11), CPVT1 iPSC-CM (*n* = 5), CPVT2 iPSC-CM (*n* = 8). In (D, F and G), the effect of ryanodine was expressed as per cent change from control. In (E), the effect of ryanodine was expressed as the change in the fluorescence ratio ΔF_340/380_, *P < 0.05, **P < 0.001. Asterisks above columns represent statistically significant effect of ryanodine; asterisk above bars connecting columns represent significant difference between groups. (H) A schematic model explaining the effects of ryanodine in the three groups. Whereas in control cardiomyocytes ryanodine acts as an antagonist and blocks SR Ca^2+^ release, in the mutated cardiomyocytes, due to altered sensitivity of the deregulated RyR2, ryanodine at 10 μM acts as an agonist, and releases SR Ca^2+^ into the cytoplasm.

Next, we determined the effects of 10 μM ryanodine on the [Ca^2+^]_i_ transient properties, which at this concentration causes a negative inotropic effect in hESC-CM and iPSC-CM 12–14 (Fig.9 and Fig. S3). The effects of ryanodine are presented as per cent (%) of change from the base line (before exposure to ryanodine) with the exception of the change in diastolic Ca^2+^ levels which is presented as ΔF_340/380_. The response to ryanodine differed markedly between control clone 24.5 and the mutated cardiomyocytes (CPVT1 clone 15.1 and CPVT2 clone 20.1; Fig.9A–C). Similar results are shown for control clone KTN3, CPVT1 clone 15.4 and CPVT2 clone 19.1 in Figure S3A–C. Whereas in control cardiomyocytes (*n* = 11) ryanodine decreased the [Ca^2+^]_i_ transient amplitude (−26.3%), +d[Ca^2+^]_i_/dt (−29.4%) and −d[Ca^2+^]_i_/dt (−15.3%) (intracellular [Ca^2+^]_i_ was unaffected), the effect on CPVT1 (*n* = 5) and CPVT2 cardiomyocytes (*n* = 8) was much more pronounced. In both CPVT1 and CPVT2 cardiomyocytes, ryanodine caused marked elevation in [Ca^2+^]_i_, along with reduction of [Ca^2+^]_i_ transient amplitude (−51.7% and −47.8%, respectively), +d[Ca^2+^]_i_/dt (−51.9 and −52.8 respectively) and −d[Ca^2+^]_i_/dt (−37.7% and −23.5%, respectively).

## Discussion

Catecholaminergic polymorphic ventricular tachycardia is a malignant inherited arrhythmia caused by mutations in the RyR2 and CASQ2 genes. Because CPVT is related to diastolic [Ca^2+^]_i_-overload, and since CASQ2 and RyR2 participate in different aspects of the E-C coupling, we hypothesized that mutations in these genes might be associated with distinct disturbances in the intracellular Ca^2+^ handing machinery. Whereas several studies 4–6,15,16 including ours 7 have used iPSC-CM to investigate mainly the arrhythmogenic outcome of CPVT1 and CPVT2, this is the first study which utilizes a variety of experimental protocols to compare the pathophysiological consequences of CASQ2 and RyR2 mutations. Our major findings were: (*i*) Ultrastructurally, CASQ2 and RyR2 mutated cardiomyocytes were less developed than control cardiomyocytes, and had a slower spontaneous firing rate. (*ii*) While in control iPSC-CM isoproterenol caused positive inotropic and lusitropic effects, in the mutated cardiomyocytes isoproterenol was either ineffective, caused arrhythmias, or markedly increased intracellular Ca^2+^, along with negative inotropic and lusitropic effects. Importantly, positive inotropic and lusitropic effects were not induced in mutated cardiomyocytes. (*iii*) The effects of caffeine and ryanodine in mutated cardiomyocytes differed from control cardiomyocytes. Our results show that iPSC-CM are useful for investigating the similarities/differences in the pathophysiological consequences of RyR2 *versus* CASQ2 mutations underlying CPVT1 and CPVT2, respectively.

### iPSC-CM generated from CPVT1 and CPVT2 patients

Thus far, 6 groups (including ours) reported on the generation of iPSC-CM from CPVT patients, and presented the basic features of the disease, including elevated intracellular Ca^2+^. Of these groups, 5 generated CPVT1 iPSC-CM from the following RyR2 mutations: F2483I 4,8, S402L 6, M4109R 5, P2328S 15 and E2311D 16, while we have generated iPSC-CM from CPVT2 patients carrying the CASQ2 D307H mutation 7. In the current study we generated iPSC-CM from the yet unstudied R420Q RyR2 mutation 10, and added 2 CPVT2 patients to the 2 patients (carrying the D307H CASQ2 mutation) we previously investigated 7,11. Importantly, including mutated iPSC-CM from both CPVT1 and CPVT2 patients enabled us to compare the pathophysiological features of the respective mutations.

The pluripotency of the reprogrammed cells was confirmed by their expression of typical pluripotent markers (Fig.2) and ability to differentiate *in vivo*, using the teratoma assay, to all three germ layers (Fig.2). The presence of the mutations R420Q in the RyR2 gene and D307H in the CASQ2 gene were verified in the iPSC derived from CPVT1 and CPVT2 patients, respectively (Fig.3). The CPVT1 and CPVT2 iPSC differentiated into functional cardiomyocytes, as demonstrated by the expression of the cardiac myofilaments proteins (α-sarcomeric actinin and troponin I) organized in sarcomeric structures and by their spontaneously beating (Fig.4). In agreement with our previous findings 7 the beating rate of both CPVT1 and CPVT2 cardiomyocytes was ∼60% slower than control cardiomyocytes. In support of these findings: (*i*) Knollmann *et al*. reported that electrocardiograms showed slower resting heart rates in both CASQ^307/307^ and CASQ2^−/−^ mice compared with WT or heterozygous mutant mice 17 and (*ii*) slower sinus heart rates are also reproducibly reported in either type of CPVT patients 18,19.

### Ultrastructural changes in CPVT1 and CPVT2 iPSC-CM

Transmission electron microscopy of control, CPVT1 and CPVT2 cardiomyocytes exhibited immature ultrastructural features represented by myofibrils with variable degrees of organization in sarcomeric pattern, poorly developed SR, high mass of glycogen and large amount of mitochondria which resemble the foetal-phenotype as previously demonstrated in cardiomyocytes generated from hESC and hiPSC 7,20,21. The CPVT1 and CPVT2 cardiomyocytes were less mature than control, having an underdeveloped ultrastructure, with shorter myofibrils, slenderer and disarrayed sarcomeres. A similar phenotype was previously reported in adult atrial myocytes obtained from auricles of cardiac patients with coronary artery disease, valvular pathology, dilated cardiomyopathy or persistent arrhythmia, which often showed thin and irregular myofilaments with widened and disarrayed Z bands 22. In our electron microscopy images, RyR complexes appeared within the SR lumen and in parallel with the SR membranes, as dense nanostructures (‘feet’) connecting the pSR to sarcolemma, and CASQ2 complexes appeared as electron-dense ‘clumps’ 17,23 or ‘chain-like’ structures 24–26 (Fig.5).

Compared to control, in CPVT1 and CPVT2 cardiomyocytes we noted a ∼80% reduction in the number of RyR complexes and absence of a dense content consistent with CASQ2 polymers, both implying damaged Ca^2+^-release unit (CRU). Furthermore, compared to control, the average width of the pSR (Table1) was narrower (*P* < 0.05) than control. In contrast, in CPVT2 the width of the pSR was similar to control (Table1, the decrease was not statistically different), but larger than CPVT1 (Table1), supporting the structural/functional interactions between CASQ and RyR2 27. For example, CASQ2 probably plays a role as a luminal senor by inhibiting RyR2 function al low SR Ca^2+^ following SR Ca^2+^ release 28. Indeed, CASQ2, RyR2 and the intrinsic membrane proteins triadin and junctin form a macromolecular complex, but their structural interaction is unknown 29. Similar morphological changes of SR were observed in cardiomyocytes from mice expressing mutant CASQ2^D307H^ 30. Furthermore, enlarged SR cisternae, either empty or containing unstructured dense material, were reported in mice models of CPVT2: CASQ2^R33Q/R33Q^ mice 23,26,27 and CASQ2^−/−^ mice 23,24,26.

### Disturbances in the Ca^2+^ handling machinery in CPVT1 and CPVT2 iPSC-CM

#### β-adrenergic stimulation

The abnormalities in the Ca^2+^ handling machinery in CPVT mutated cardiomyocytes can be unmasked/deciphered by analysing the responsiveness to β-adrenergic stimulation, which affects several aspects of the E-C coupling. Collectively, in CPVT (with no difference between CPVT1 and CPVT2) cardiomyocytes the response to isoproterenol could be divided to three groups. The first group included cardiomyocytes (59% in CPVT1 and 37% in CPVT2) which were completely unresponsive to isoproterenol, including lack of arrhythmias. The second group included cardiomyocytes (16% in CPVT1 and 37% in CPVT2) in which isoproterenol generated triggered beats, yet the [Ca^2+^]_i_ transient characteristics were unaffected. In the third group (25% in CPVT1 and 26% in CPVT2) isoproterenol markedly increased diastolic [Ca^2+^]_i_, along with a pronounced decline in +d[Ca^2+^]_i_/dt and −d[Ca^2+^]_i_/dt, corresponding to negative inotropic and lusitropic effects, respectively. While a single mechanism is unlikely to account for the three different responses to isoproterenol in the mutated cardiomyocytes (Figs6 and 7), the ultrastructural findings may at least in part, account for the abnormal β-adrenergic responsiveness. Specifically, the analysis showed (Fig.5) that in both CPVT1 and CPVT2 cardiomyocytes, the number of RyR2 units was reduced by ∼80% compared to control cardiomyocytes, which can contribute to the lack of positive inotropic and lusitropic effects of isoproterenol. Additionally, mutated RyR2 were shown to be associated with abnormal sympathetic response, although the relevance to this study has not been demonstrated. For example, Shan *et al*. showed that RyR^2S2808A+/+^ mice harbouring RyR2 channels that cannot be PKA phosphorylated, exhibited blunted heart rate and cardiac contractile responses to isoproterenol. Furthermore, the typical isoproterenol-induced increase of ventricular myocyte [Ca^2+^]_i_ transient amplitude and contraction were all attenuated in the RyR^2S2808A+/+^ mice 31.

In the third group in which isoproterenol increased intracellular Ca^2+^, all 3 [Ca^2+^]_i_ transient characteristics were markedly attenuated (Fig.7). Three putative mechanisms can account for the attenuated [Ca^2+^]_i_ transients in the presence of increased intracellular Ca^2+^. (*i*) Augmented diastolic SR Ca^2+^ leak diminishes SR Ca^2+^ reservoir, and therefore less RyR2-mediated Ca^2+^ efflux into the cytoplasm. (*ii*) Ca^2+^-induced inhibition of the L type Ca^2+^ current (I_Ca,L_) 32–34 will reduce I_Ca,L_ density, thereby attenuating [Ca^2+^]_i_ transient amplitude and +d[[Ca^2+^]_i_/dt_max_. (*iii*) The excessive rise in intracellular Ca^2+^ diminishes the Ca^2+^ gradient across the SR membrane and therefore the driving force for Ca^2+^ efflux into the cytoplasm, which in turn will attenuate the [Ca^2+^]_i_ transient. The emanating question from these findings is why isoproterenol generated three distinct phenotypes in a seemingly similar population of EBs. We hypothesize that these diverse effects could be related to the heterogeneous composition of the EBs regarding the ratio between nodal, atrial and ventricular cardiomyocytes, including Purkinje fibres. In this regards, based on optical mapping in isolated Langendorff-perfused RyR^R4496C/WT^ CPVT mouse hearts, Jalife’s group suggested that CPVT arrhythmias originate from the Purkinje fibres 35, probably because of their Ca^2+^ handling characteristics, which promote spontaneous Ca^2+^ release and triggered arrhythmias 36–38. Finally, an intriguing observation was that in mutated cardiomyocytes in which isoproterenol induced arrhythmias intracellular Ca^2+^ was unaffected, and in cells where Ca^2+^ levels were elevated, arrhythmias were not generated. This distinction between the two effects of isoproterenol suggests that the triggered beats originated delayed afterdepolarizations (DADs) caused by spontaneous diastolic SR Ca^2+^ release (SOICR, SR Ca^2+^ overload) and not due to cytoplasmic Ca^2+^ overload although this phenomena does occur in CPVT cardiomyocytes.

### The responsiveness of CPVT1 and CPVT2 to caffeine and ryanodine

#### The effects of caffeine in CPVT cardiomyocytes

The caffeine and ryanodine experiments further elucidated the abnormal functionality of internal Ca^2+^ stores in CPVT cardiomyocytes, and distinguished between CPVT1 and CPVT2. In brief, while in CPVT1 cardiomyocytes caffeine released less Ca^2+^ (represented by the change in [Ca^2+^]_i_ transient amplitude and area) than in control cardiomyocytes, in CPVT2 cardiomyocytes the release of Ca^2+^ from intracellular stores was markedly augmented (Fig.8). Furthermore, although control and CPVT1 cardiomyocytes displayed rapid recovery after caffeine-induced SR Ca^2+^ release, in CPVT2 cardiomyocytes, the recovery towards pre-caffeine levels was much slower. Recent studies provide some support for these findings. Kujala *et al*. found that compared to control, caffeine-induced Ca^2+^ release was smaller in CPVT1 iPSC-CM (RyR2^P2328S^) 15, in agreement with our results. Similarly, Zhang *et al*. reported that caffeine-induced Ca^2+^ transients generated smaller NCX current in CPVT1 iPSC-CM (RyR2^F24831^), compared to control, reflecting their smaller Ca^2+^ stores 8. As proposed in the scheme shown in Figure8F, the effects of caffeine in CPVT1 and CPVT2 cardiomyocytes reflect the SR Ca^2+^ storage capacity as well as the mutation phenotype. Accordingly, we suggest that the massive and prolonged Ca^2+^ release in CPVT2 cardiomyocytes originated from high levels of free SR Ca^2+^ due to the mutated CASQ2 protein [*e.g*. 3], and not necessarily from a total higher SR Ca^2+^ levels. In contrast, the attenuated caffeine-induced Ca^2+^ release in CPVT1 cardiomyocytes is probably due to the mutated RyR2, which is likely to have low sensitivity to caffeine along with reduced SR Ca^2+^ storage, as suggested by the ultrastructural analysis in which CPVT1 iPSC-CM presented decreased SR peripheral width (−15%) compared to control cardiomyocytes.

#### The effects of ryanodine in CPVT cardiomyocytes

In control cardiomyocytes 10 μM ryanodine attenuated the [Ca^2+^]_i_ transient amplitude and area (−26.26% and −27.85%, respectively), in agreement with previous studies in iPSC-CM and hESC-CM 12,13,39 and in rabbit cardiomyocytes 40, but did not affect diastolic [Ca^2+^]_i_. In contrast, in CPVT cardiomyocytes ryanodine markedly elevated diastolic [Ca^2+^]_i_ along with pronounced decline in the [Ca^2+^]_i_ transient characteristics. These results can be explained based on the scheme shown in Figure9H; whereas in control cardiomyocytes ryanodine acts as an antagonist and blocks SR Ca^2+^ release, in the mutated cardiomyocytes, due to altered sensitivity of the deregulated RyR2, ryanodine at 10 μM acts as an agonist and releases SR Ca^2+^ into the cytoplasm.

In summary, for the first time we compared iPSC from CPVT1-RyR2^R420Q^ and CPVT2-CASQ2^D307H^ patients, which gave rise to functional mutated cardiomyocytes exhibiting the disease-specific arrhythmogenic characteristics. This experimental system enables us to recapitulate *in vitro* the features of human genetic disorders, such as CPVT, in which the disease mechanisms can be explored and candidate drugs screened and developed in the physiologic and disease-causing settings on a patient-specific level. Indeed, both CPVT1 and CPVT2 cardiomyocytes generated arrhythmias in response to β-adrenergic stimulation and displayed Ca²^+^ abnormalities in response to ryanodine. The exposure of the mutant iPSC-CM to caffeine revealed the different intracellular Ca²^+^ abnormalities between CPVT1 and CPVT2 cardiomyocytes. Collectively, our results support the notion that the propensity to develop arrhythmias in CPVT is associated with disturbed intracellular Ca^2+^ handling, SR load and storage capacity.

## Conflicts of interest

The author confirm that there are no conflicts of interest.

## Supporting Information

**Data S1** Methods and additional results.
