# Supplementary material for: Functional abnormalities in iPSC-derived cardiomyocytes generated from CPVT1 and CPVT2 patients carrying ryanodine or calsequestrin mutations
Source: J Cell Mol Med. 2015 Jul 8;19(8):2006–18. doi: 10.1111/jcmm.12581 (PMC4549051; doi:10.1111/jcmm.12581)
Supplement: Supplementary file 1 [file jcmm0019-2006-sd1.pdf]

SUPPLEMENT

Functional abnormalities in iPSC-derived cardiomyocytes generated from CPVT1 and CPVT2 patients carrying ryanodine or calsequestrin mutations

[a,b,c]Atara Novak*,[a,b,c]Lili Barad*, [c,d]Avraham Lorber, [e]Mihaela Gherghiceanu, [a,b,c]Binyamin Eisen, [f]Liron Eldor, [b,c]Joseph Itskovitz-Eldor, [g]Michael Eldar, [g]Michael Arad, [a,b,c]Ofer Binah

[a]Department of Physiology, [b]The Rappaport Institute for Research in the Medical Sciences, [c]Ruth & Bruce Rappaport Faculty of Medicine, Technion, Haifa, Israel. [d]Department of Pediatric Cardiology, Rambam Health Care Campus, Haifa, Israel. [e]'Victor Babes' National Institute of Pathology, Bucharest, Romania. [f]Department of Plastic Surgery, Rambam Health Care Campus, Haifa, Israel. [g]Leviev Heart Center, Sheba Medical Center, Tel Hashomer and Sackler School of Medicine, Tel Aviv University, Tel Aviv, Israel.

Methods

Generation of patient-specific iPSC

Skin biopsies were obtained from a 25-year old CPVT1 woman (HDF15) carrying the R420Q mutation in the RyR2 gene, and from 4 CPVT2 patients carrying the D307H mutation in the CASQ2 gene: 12-year old boy (HDF7), 30-year old woman (HDF12) and two brothers at the age of 18 (HDF19) and 20 (HDF20). As controls we used a skin biopsy from a 46-year old woman (HDF24), and hair keratinocytes from the plucked hairs of 2 healthy women at the age of 36 (KTN) and 41 (KTR), as previously reported [1]. All donors signed consent forms according to approval # 3116 by the Helsinki Committee for experiments on human subjects at Rambam Health Care Campus, Haifa, Israel. iPSC were generated from human dermal fibroblasts and hair keratinocytes using the STEMCCA Cassette (a single lentiviral vector containing the 4 factors: oct4, sox2, klf4 and c-myc) as previously described [1-3] or by the CytoTune™-iPS Reprogramming kit according to the manufacture instructions (Invitrogen, Grand Island, NY, USA). From the human dermal fibroblasts we used: CPVT1 clones 15.1 and 15.4 from biopsy HDF15; CPVT2 clones 7.5, 7.6, 12.4, 19.1, 19S1, 20.1 and 20S3 from biopsies HDF7, HDF12, HDF19 and HDF20, respectively; control clones 24.3 and 24.5 from biopsy HDF24. From hair keratinocytes we used the control clones KTN3 and KTR13 from the KTN and KTR biopsies, respectively. Clones 19S1 and 20S3 were generated using the Sendai virus reprogramming kit, and all other clones were generated using the STEMCCA cassette.

Genotyping and karyotyping analyses

Genomic DNA was purified using the Promega (Madison, WI, USA) DNA purification kit. To confirm that the mutation was preserved in the iPSC clones, we performed PCR reaction to genomic DNA with primers that delimit the mutation area on the RyR2 or the CASQ2 gene. PCR was performed to the RyR2 gene using the primers: F-5'-TGGCTCAGCTGTTTGAGTACA-3' and R-5'-TCTCTCGCAAGTCAGAGCAT-3' resulting in a product length of 990 nucleotides and to the CASQ2 gene using the primers: F-5'-CACTCTGCTCTCCACATTAGAAGCTGT-3' and R-5'-AAAAGTAGTTCTGGGGACTGGGAATGG-3' resulting in a product length of 495 nucleotides. Karyotype analysis was performed using standard G-banding chromosome analysis by the cytogenetic laboratory according to standard procedures.

Immunofluorescence analyses

Immunofluorescence staining was performed according to standard protocols using the following antibodies: rabbit anti-Oct3/4 (1:100, Santa Cruz, Dallas, Texas, USA), goat anti-nanog (1:20, R&D), mouse anti-Sox2 (1:100, Millipore), mouse anti-TRA 1-60 (1:100, Millipore, Billerica, MA, USA), mouse anti-TRA 1-81 (1:100, Millipore), mouse anti-SSEA4 (1:100, Millipore), rabbit anti-cardiac troponin I (1:400, Abcam), mouse anti-sarcomeric α-actinin (1:600, Sigma-Aldrich, St. Louis, MO, USA). Secondary antibodies were as follows: donkey anti-rabbit Cye 3 (1:100, Invitrogen) and donkey anti-mouse/goat Alexafluor 488 (1:100, Invitrogen). Cells were also stained with DAPI (1:1000, Boehringer, Germany) for nuclei detection.

Measurements of $[Ca^{2+}]_i$ transients

$[Ca^{2+}]_i$ transients were measured from small contracting areas of EBs by means of fura-2 fluorescence as previously described [1, 4]. Spontaneously contracting areas of EBs with a size range of 0.5-1 mm were mechanically dissected and adhered onto 18 mm diameter gelatin coated glass slides. Subsequently, fura-2 stained contracting areas were transferred to a chamber mounted on heated (37°C) stage of an inverted microscope and perfused with Tyrode's solution at 37°C. The preparations were paced at a frequency 10% higher than the spontaneous rate, constituting pacing frequencies in the range of 0.5–1 Hz. The acquisition rate of both the $[Ca^{+2}]_i$ transients and contractions was 100 points/sec. Using a dedicated MATLAB package, the following $[Ca^{2+}]_i$ transient characteristics were analyzed and averaged in 10 successive contractions. The $[Ca^{2+}]_i$ transient amplitude was calculated as the difference between the maximal (systolic) and the minimal (diastolic) fluorescence ratio. Maximal rates of $[Ca^{2+}]_i$ increase ($+d[Ca^{2+}]_i/dt$) and decrease ($-d[Ca^{2+}]_i/dt$) were represented by the maximal slopes of the ascending and descending limbs of the $[Ca^{2+}]_i$ transient signal. Data were presented as mean of change from the base line in % ± standard error of mean (SEM) with the exception of the change in diastolic Ca^{2+} levels which was presented as mean $\Delta F_{340/380}$ ± SEM.

Transmission electron microscopy

Transmission electron microscopy (TEM) was performed on 60-62-day-old (post-plating) EBs which were fixed with 2.5% glutaraldehyde in 0.1M cacodylate buffer [pH 7.4, room temperature (RT)], and post-fixed for 1 hr in buffered 1% OsO4 with 1.5% K4Fe(CN)6 (potassium ferrocyanide-reduced osmium) at RT. Fixed EBs were embedded in 1% agar, dehydrated in graded ethanol series and further processed for epoxy resin (Agar100) embedded

at 60°C for 48 hrs. The ultra-thin sections were cut with a diamond knife at 60 nm thicknesses using an RMC ultramicrotome (Boeckeler Instruments Inc., Tucson, AZ, USA) and double stained with 1% uranyl acetate and Reynolds's lead citrate. Ultrastructural examination was performed with a Morgagni 268 transmission electron microscope (FEI Company, Eindhoven, The Netherlands) at 80 kV. Digital electron micrographs were recorded with a MegaView III CCD and iTEM-SIS software (Olympus, Soft Imaging System GmbH, Münster, Germany) was used for morphometry.

Statistical analysis

The results are presented as Mean±SEM and the significance of difference between the groups was evaluated using Two Way ANOVA followed by Holm-Sidak test except TEM analysis which was evaluated using One Way ANOVA followed by Dunn's test. $P<0.05$ was considered statistically significant, where (*) represents $P<0.05$ and (**) represents $P<0.001$.

Results

Ca^{2+} handling abnormalities in mutated iPSC-CM

β-adrenergic stimulation

Figure 1 depicts representative $[Ca^{2+}]_i$ transients in control versus mutated iPSC-CM in response to isoproterenol. In control iPSC-CM clones KTN3 and KTR13 (Fig. 1A) isoproterenol increased (as expected) the $[Ca^{2+}]_i$ transient amplitude, the maximal rate of rise and decay ($+d[Ca^{2+}]_i/dt$ and $-d[Ca^{2+}]_i/dt$, respectively), while diastolic $[Ca^{2+}]_i$ was unchanged. In contrast, in CPVT1 iPSC-CM and CPVT2 iPSC-CM isoproterenol caused 3 dissimilar types of effects (Fig.

1B-D): (1) "No-response" to isoproterenol (Fig. 1B, CPVT1 iPSC-CM clone 15.1 and CPVT2 iPSC-CM clones 19S1 and 12.4). (2) "Arrhythmias" (Fig. 1C, CPVT1 iPSC-CM clone 15.1 and CPVT2 iPSC-CM clones 19S1, 12.4 and 7.5). (3) "Intracellular Ca^{2+} rise" associated with diminished $[Ca^{2+}]_i$ transient amplitude (Fig. 1D, CPVT1 iPSC-CM clone 15.1 and CPVT2 iPSC-CM clones 20.1 and 7.5).

Responsiveness to caffeine

Fig. 2 depicts representative Ca^{2+} storage/release capacities of the mutated versus control cardiomyocytes, by rapid application of 10 mM caffeine (an opener of the RyR2 receptor) to paced cardiomyocytes. In control iPSC-CM clone 24.5 (Fig. 2A) caffeine caused an abrupt increase in intracellular Ca^{2+} along with a sharp decline in the $[Ca^{2+}]_i$ transient amplitude. Within 20 sec after intracellular Ca^{2+} picked, it declined and the $[Ca^{2+}]_i$ transients attained a steady state level close to the their pre-caffeine amplitude. In CPVT1 iPSC-CM clone 15.4 (Fig. 2B) the response to caffeine was smaller and shorter, and the resumption of pre-caffeine intracellular Ca^{2+} level and $[Ca^{2+}]_i$ transient amplitude was much faster than in control cardiomyocytes. In sharp contrast, in CPVT2 iPSC-CM clone 19S1 (Fig. 2C) the response to caffeine was markedly augmented.

Responsiveness to ryanodine

Fig. 3 shows representative $[Ca^{2+}]_i$ transients in control versus mutated iPSC-CM in response to 10 µM ryanodine. The response to ryanodine differed markedly between control

clone KTN3 and the mutated cardiomyocytes (CPVT1 iPSC-CM clone 15.4 and CPVT2 iPSC-CM clone 19.1, Fig. 3A-C). Whereas in control cardiomyocytes ryanodine decreased the $[Ca^{2+}]_i$ transient amplitude, $+d[Ca^{2+}]_i/dt$ and $-d[Ca^{2+}]_i/dt$, (intracellular $[Ca^{2+}]_i$ was unaffected), the effect in CPVT1 and CPVT2 cardiomyocytes was much more pronounced. In both CPVT1 and CPVT2 cardiomyocytes ryanodine caused marked elevation in $[Ca^{2+}]_i$, along with reduction of $[Ca^{2+}]_i$ transient amplitude, $+d[Ca^{2+}]_i/dt$ and $-d[Ca^{2+}]_i/dt$.

Supplement Figure Legends

Figure 1

The effects of isoproterenol on the $[Ca^{+2}]_i$ transients in control, CPVT1and CPVT2 iPSC-CM. (A) $[Ca^{+2}]_i$ transients from control (clones KTN3 and KTR13) iPSC-CM, in the absence and presence of isoproterenol. (B - D) Representative experiments in CPVT1 and CPVT2 iPSC-CM demonstrating the 3 types of responses to isoproterenol. (B) No response to isoproterenol. CPVT1 iPSC-CM clone 15.1 day 50, CPVT2 iPSC-CM clones 19S1 and 12.4. (C) Isoproterenol induced triggered beats. CPVT1 iPSC-CM clone 15.1, CPVT2 iPSC-CM clones 19S1, 12.4 and 7.5. (D) Isoproterenol increased diastolic $[Ca^{+2}]_i$. CPVT1 iPSC-CM clone 15.1, CPVT2 iPSC-CM clones 20.1 and 7.5. In (A-D) the pacing rates are shown in each panel.

Figure 2

The effect of caffeine on the intracellular Ca^{2+} cycling of control, CPVT1 and CPVT2 iPSC-CM. (A - C): $[Ca^{2+}]_i$ transients from control (clone 24.5), CPVT1 (clone 15.4) and CPVT2 (clone 19S1) iPSC-CM, respectively demonstrating the effect of caffeine.

Figure 3

The effects of ryanodine on the $[Ca^{2+}]_i$ transients of control, CPVT1 and CPVT2 iPSC-CM. (A-C): $[Ca^{2+}]_i$ transients from the 3 groups demonstrating the effect of ryanodine in Control iPSC-CM clone KTN3, CPVT1 iPSC-CM clone 15.4 and CPVT2 iPSC-CM clone 19.1, respectively.

References

1. **Novak A, Shtrichman R, Germanguz I, *et al***. Enhanced reprogramming and cardiac differentiation of human keratinocytes derived from plucked hair follicles, using a single excisable lentivirus. *Cell Reprogram*. 2010; 12: 665-78.
2. **Somers A, Jean JC, Sommer CA, *et al***. Generation of transgene-free lung disease-specific human iPS cells using a single excisable lentiviral stem cell cassette. *Stem Cells*. 2010.
3. **Sommer CA, Stadtfeld M, Murphy GJ, *et al***. Induced pluripotent stem cell generation using a single lentiviral stem cell cassette. *Stem Cells*. 2009; 27: 543-9.
4. **Dolnikov K, Shilkrut M, Zeevi-Levin N, *et al***. Functional properties of human embryonic stem cell-derived cardiomyocytes: intracellular Ca^{2+} handling and the role of sarcoplasmic reticulum in the contraction. *Stem Cells*. 2006; 24: 236-45.

Figure 1

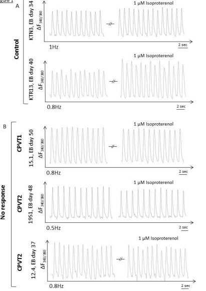
A
Control
KTN3, EB day 34
ΔF340/380
1 μM Isoproterenol
1Hz
2 sec
KTR13, EB day 40
ΔF340/380
1 μM Isoproterenol
0.8Hz
2 sec
B
No response
CPVT1
15.1, EB day 50
ΔF340/380
1 μM Isoproterenol
0.8Hz
2 sec
CPVT2
1951, EB day 48
ΔF340/380
1 μM Isoproterenol
0.5Hz
2 sec
CPVT2
12.4, EB day 37
ΔF340/380
1 μM Isoproterenol
0.8Hz
2 sec

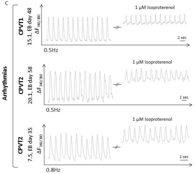
C
Arrhythmias
CPVT1
15.1, EB day 48
ΔF 340/380
1 μM Isoproterenol
2 sec
0.5Hz
CPVT2
20.1, EB day 58
ΔF 340/380
1 μM Isoproterenol
2 sec
0.5Hz
CPVT2
7.5, EB day 35
ΔF 340/380
1 μM Isoproterenol
2 sec
0.8Hz

D

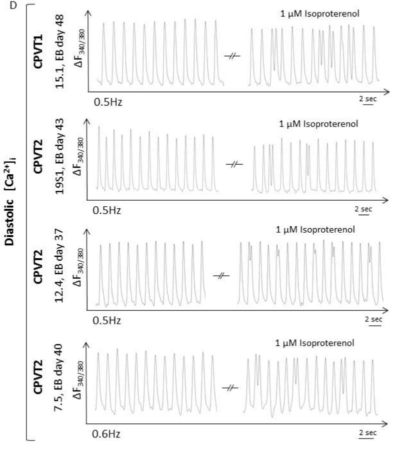

Diastolic [Ca2+]i
CPVT1
15.1, EB day 48
ΔF340/380
0.5Hz
1 μM Isoproterenol
2 sec
CPVT2
19S1, EB day 43
ΔF340/380
0.5Hz
1 μM Isoproterenol
2 sec
CPVT2
12.4, EB day 37
ΔF340/380
0.5Hz
1 μM Isoproterenol
2 sec
CPVT2
7.5, EB day 40
ΔF340/380
0.6Hz
1 μM Isoproterenol
2 sec

Figure 2

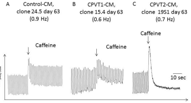

A
Control-CM,
clone 24.5 day 63
(0.9 Hz)
Caffeine
B
CPVT1-CM,
clone 15.4 day 63
(0.6 Hz)
Caffeine
C
CPVT2-CM,
clone 19S1 day 63
(0.7 Hz)
Caffeine
10 sec
ΔF 340/380

Figure 3

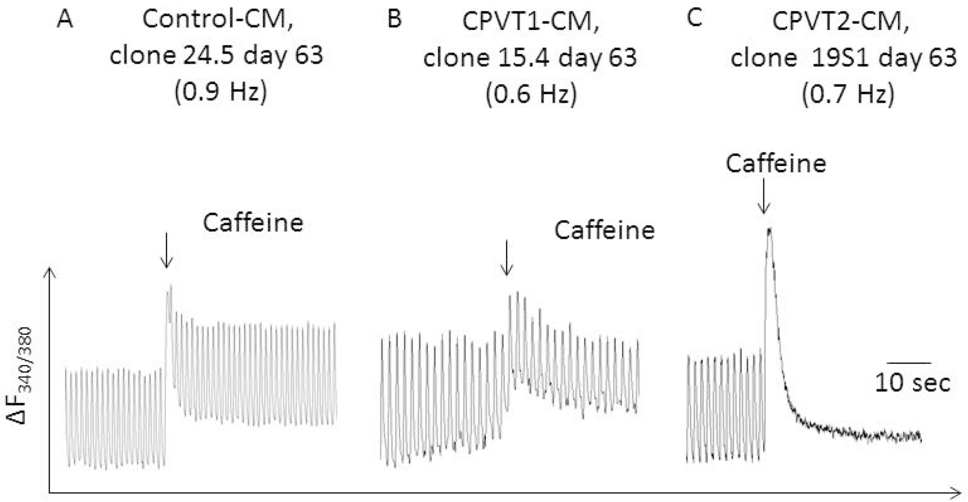
A
Control-CM,
clone 24.5 day 63
(0.9 Hz)
B
CPVT1-CM,
clone 15.4 day 63
(0.6 Hz)
C
CPVT2-CM,
clone 19S1 day 63
(0.7 Hz)
Caffeine
Caffeine
Caffeine
ΔF340/380
10 sec
